# Adsorptive Removal of Antibiotic Ciprofloxacin from Aqueous Solution Using Protein-Modified Nanosilica

**DOI:** 10.3390/polym12010057

**Published:** 2020-01-01

**Authors:** Tien Duc Pham, Thi Ngan Vu, Hai Long Nguyen, Pham Hai Phong Le, Thi Sim Hoang

**Affiliations:** 1 Faculty of Chemistry, VNU University of Science, Vietnam National University, Hanoi, 19 Le Thanh Tong, Hoan Kiem, Hanoi 100000, Vietnam; vuthingan_t62@hus.edu.vn (T.N.V.); simhoang.hus@gmail.com (T.S.H.); 2 HUS High School for Gifted Students, VNU University of Science, Vietnam National University, Hanoi, 182 Luong The Vinh, Thanh Xuan, Hanoi 100000, Vietnam; ceonguyenhailong@gmail.com (H.L.N.); lephamhaiphong@gmail.com (P.H.P.L.)

**Keywords:** ciprofloxacin, adsorption, protein, moringa seeds, nanosilica, two-step model

## Abstract

The present study aims to investigate adsorptive removal of molecular ciprofloxacin using protein-modified nanosilica (ProMNS). Protein was successfully extracted from Moringa seeds while nanosilica was synthesized from rice husk. Fourier-transform infrared (FTIR), ultraviolet visible (UV-Vis) and high-performance liquid chromatography (HPLC) were used to evaluate the characterization of protein. Adsorption of protein onto nanosilica at different pH and ionic strength was thoroughly studied to modify nanosilica surface. The removal efficiency of antibiotic ciprofloxacin (CFX) increased from 56.84% to 89.86% after surface modification with protein. Effective conditions for CFX removal using ProMNS were systematically optimized and found to be pH 7.0, adsorption time 90 min, adsorbent dosage 10 mg/mL, and ionic strength 1 mM KCl. A two-step model was successfully used to fit the adsorption isotherms of CFX onto ProMNS at different ionic strength while a pseudo-second-order model could fit adsorption kinetic of CFX onto ProMNS very well. Maximum adsorption capacity was very high that reached to 85 mg/g. Adsorption of CFX onto ProMNS decreased with increasing KCl concentration, suggesting that adsorption of CFX onto ProMNS is mainly controlled by electrostatic attraction between positively charged ProMNS surface and anionic species of CFX. Adsorption mechanisms of CFX onto ProMNS were discussed in detail based on adsorption isotherms, the change in surface charge by zeta potentail and the change in functional groups by FT-IR. The removal of CFX after three regenerations was greater than 73% while CFX removal from an actual hospital wastewater using ProMNS reached to 70%. Our results suggest that ProMNS is a new and eco-friendly adsorbent to remove antibiotics from aqueous solutions.

## 1. Introduction

Antibiotics, which are synthetic chemicals or natural products, are a powerful medicine to bacterial infections. Ciprofloxacin (CFX), which is one of the second generated group of synthetic Fluoroquinolones (FQs), is an important antibiotic for the treatment of both gram-negative and positive bacterial infections. The CFX makes a large broader antibacterial spectrum so that it can exhibit greater intrinsic antibacterial activity [1,2,3]. The CFX can also make a resonance with other antibiotic groups such as beta-lactam or aminoglycosid that have very high performance in disease treatment. Nevertheless, one unexpected thing is that CFX resistance is rapidly increasing to seriously high levels in developing countries. The antibiotic residual including CFX is also very toxic to organisms in an aquatic environment although the concentrations are low [4]. 

Removal of antibiotic is important for environmental concern, because antibiotic residual in aqueous solution can cause antibiotic resistance. Various techniques have been developed and used for antibiotic removal, such as membrane process [5,6,7], photocatalytic degradation [8], advanced oxidation [9], and adsorption [10,11,12]. Many studies indicate that adsorption is one of the most effective and suitable method for antibiotics removal toward green chemistry by using low eco-friendly adsorbents [13,14].

Protein is a mixture of various amino acids that is an origin of life [15,16]. Protein adsorption onto particle surface plays an important role in the biological environment so that numerous researches on protein adsorption onto solid surface have been investigated [17]. Conformation of stable protein on a negatively charged surface has been found, demonstrating that protein adsorption onto particles can be applied to modify a solid surface [18]. It is reported that Moringa (MO) seeds contain a high amount of protein, which have anti-macrobial potential [19]. Protein extracted from MO seeds as an effective coagulant reagent for water treatment was thoroughly studied [20,21]. Nevertheless, application of adsorption of protein MO seeds for antibiotic removal has not been reported. 

Silica, which is one of the most popular oxide in nature, is basically negatively charge. Silica is also easily synthesized from agricultural sub-product rice husk [22]. Due to the low charge density and non-porous structure of nanosilica from rice husk, the low adsorption of antibiotic onto nanosilica is evident. Surface modification of nanosilica by protein adsorption may be useful to remove of antibiotic. Many studies investigated adsorption different kinds of proteins onto silica to find the characteristics of protein on silica surface [15,18]. However, protein adsorption onto nanosilica to enhance removal of antibiotic CFX has not been studied.

An understanding of adsorption mechanisms of antibiotic onto protein-modified nanosilica (ProMNS) is needed for further application to a real system in the presence of many interferences. For this purpose, adsorption isotherms by different modes is often evaluated. The two-step model proposed by Zhu et al. [23], with a general adsorption isotherm equation, was successfully applied to various types of antibiotic adsorbates for numerous systems [4,24,25]. Additionally, the two-step model is able to evaluate the growth of adsorbed layers [26,27]. Therefore, the two-step model is suitable to fit adsorption isotherms of CFX onto ProMNS. 

In this paper, for the first time, we report adsorptive removal of CFX using nanosilica fabricated from rice husk with surface modification by adsorption of MO seeds protein. The effective conditions on CFX removal were systematically studied. Adsorption mechanisms are suggested based on surface modification by Fourier transform infrared spectroscopy (FTIR), the change in surface charge by ζ potential measurements, and adsorption isotherm. The application of the optimum condition for CFX removal for actual hospital wastewater is also investigated in the present study.

## 2. Experimental

### 2.1. Materials

**Figure 1:**
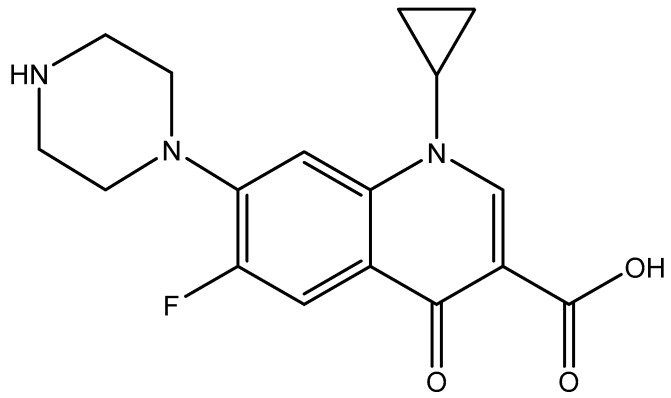
Chemical structure Cirofloxacin (CFX).

The Moringa (MO) seeds used in the present study were purchased from Minh Tue Company (Hanoi, Vietnam) while rice husk was collected from Bacninh, Vietnam. Nanosilica was synthesized and characterized by the hydrothermal method according to our previously published papers [4,22,24]. Ciprofloxacin hydrochloride monohydrate (CFX) (CAS 86393-32-0) with purity higher than 98% (HPLC grade) was supplied by Tokyo Chemical Industry (Tokyo, Japan). The chemical structure of CFX is shown in Figure 1. Solid ammonium sulfate (NH_4_)_2_SO_4_ and acetone to synthesize protein was purchased from Scharlau (Spain, EU). Amino acid standards were acquired from Sigma Aldrich (St. Louis, MO, USA). Methanol and Acetonitrile for HPLC were supplied from Merck. The KCl, HCl, and KOH (p.A, Merck) were used to study the effect of ionic strength and adjust pH solution. Solution pH was measured using an HI 2215 pH meter (Hanna, Woonsocket, RI, USA). Before use, the pH electrode was calibrated with three standard buffers of 4.01, 7.01, and 10.01 (Hanna). Other chemicals with analytical grade were also supplied from Merck. An ultrapure water produced from the ultrapure water system (Labconco, Kansas City, MO, USA) with a resistivity of 18.2 MΩcm was used to prepare all aqueous solutions.

### 2.2. Fabrication and Purification of Protein from Moringa Seeds

**Figure 2:**
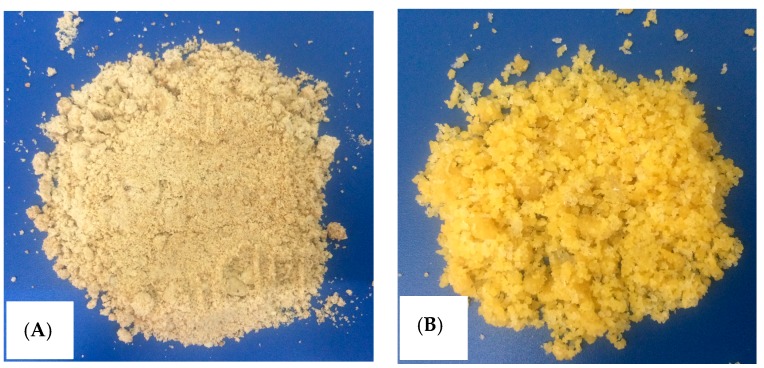
Photos of (**A**) milled Moringa seeds, and (**B**) synthesized protein.

Protein was extracted from Moringa (MO) seeds according to Kwaambwa et al. [16,20,28], with a modification. The MO seeds were shelled before drying at 40 °C for one week and milling to powder by mortar and pestle (Figure 2A). About 50.0 g of milled MO seed powder was mixed and extracted by with 100 mL petroleum ether to remove oil for 10 h using Orbital shaker. The extraction process was repeated 4 times with every 50 mL petroleum ether. The solids were dissolved in 200 mL pure water and filtered by filtration paper. To remove MO oil completely, liquid–liquid extraction was repeated 3 times with every 10 mL petroleum ether. Then, protein in aqueous filtration was precipitated by slowly adding solid (NH_4_)_2_SO_4_. Protein was obtained by centrifuging. Finally, powder of protein was formed with acetone before freeze-drying (Figure 2B). Purification of protein was conducted with dialysis using cellulose membrane (Sigma Aldrich). The lyophilized protein powder was kept at a fridge in a dark glass bottle. 

### 2.3. Characterization and Analytical Methods

The protein synthesized from Moringa (MO) seeds was characterized by ultraviolet-visible spectroscopy (UV-Vis), Fourier transform infrared spectroscopy (FT-IR), and high-performance liquid chromatography (HPLC) with fluorescence detection (FLD) and photo diode array detection (PDA).

The UV-Vis measurements were carried out by a double-beam spectrophotometer using a couple of quartz cuvettes with a 1 cm optical path length using (UV-1650 PC, Shimadzu, Japan). 

The FTIR spectra were conducted with an Affinity-1S spectrometer (Shimadzu, Japan). All spectra were obtained at a resolution of 4 cm^−1^ and at 25 °C and atmospheric pressure.

The HPLC-FLD and HPLC-PDA with two columns were used to quantify amino acids in MO seeds protein [29,30]. Amino acids were hydrolyzed by 6 M NaOH for 24 h at 125 °C. Then, the samples adjusted to neutral pH by HCl. After that, the solutions were filtered and diluted appropriated before injecting into HPLC systems. The reversal phase column RP18 AccQ Tag (150 mm × 4.6 mm × 3.9 µm) (Water, Milford, MA, USA) and mobile phase using acetate phosphate buffer (pH 5.05) and acetonitrile with gradient elution were used. The total time for separation with a flow rate of 1.0 mL/min was 23 min. Eluted peaks were monitored by PDA detector with a wavelength of 260 nm [30]. To quantify Tryptophan, the column Xbridge (150 mm × 4.6 mm × 5 µm) (Water, Milford, MA, USA) and mobile phase using H_2_O:acetonitrile (*v*/*v*: 95:5) with isocratic elution were used. The excited wavelength and emission wavelength were λex = 295 nm and λem = 345 nm, respectively [31].

The surface charges of nanosilica, protein-modified nanosilica (ProMNS), and ProMNS after CFX adsorption were examined by zeta (ζ) potential measurements. The ζ potential was calculated from electrophoretic mobility with Smoluchowski’s equation [32]:

(1) $$\zeta =\frac{u_{e}\;\eta }{\varepsilon_{rs}\;\varepsilon_{0}}$$

where ζ is the zeta potential (mV), $u_{e}$ the electrophoretic mobility (µms^−1^/Vcm^−1^), η is the dynamic viscosity of the liquid (mPa s), $\varepsilon_{rs}\;\mathrm{is}\;$ the relative permittivity constant of the electrolyte solution, and $\varepsilon_{0}$ is the electric permittivity of vacuum (8.854 × 10^−12^ F/m). 

### 2.4. Adsorption Studies

All adsorption experiments were conducted by batch mode in 15 mL Falcon tubes at 25 ± 2 °C controlled by an air conditioner. Initial amount of protein was precisely weighed and then diluted with ultrapure water to a stock solution of 2500 mg/L. Then, the stock solution was appropriately diluted to prepare a series of protein concentration for protein adsorption isotherms. 

A 10 mg/mL nanosilica was mixed in 10 mL of protein in the range of 50 to 2600 mg/L for 2 h. After that, the protein solutions were separated using ultra-centrifuging at 12,000 rpm (5 °C) for 10 min with a refrigerated centrifuge (MR23i, JOUAN, France). The effects of pH and ionic strength on adsorption of protein were systematically studied. The concentrations of protein were quantified by UV-Vis spectroscopy and HPLC-PDA. The adsorption capacity Γ (mg/g) of protein or CFX onto nanosilica, ProMNS was calculated by Equation (2):

(2) $$\Gamma =\frac{C_{i}-C_{f}}{m}$$

where *C_i_* (mg/L) and *C_f_* (mg/L) are the initial and the final concentrations of protein or CFX, respectively, while *m* (mg/mL) is adsorbent dosage.

For adsorptive removal of CFX, a different adsorbent dosage was mixed well with 10 mL protein using an orbital shaker orbital OS-350D (Digisystem laboratory, Taiwan) under optimum conditions to modify the nanosilica surface. The adsorbent was then washed with ultrapure water before adding the concentrations of CFX. The concentrations of CFX were determined by UV–Vis spectroscopy. The relationship between the concentrations of CFX and measured absorbance and at a wavelength of 272 nm as standard calibration curves in different conditions with a correlation coefficient of at least 0.999 was confirmed. 

The removal (% R) of CFX was calculated by Equation (3):

(3) $$Removal\;\left(\%R\right)=\frac{C_{i}-C_{e}}{C_{i}}\times 100\%.$$

The adsorption isotherms of CFX onto ProMNS were fitted by two-step model using a general isotherm equation that was successfully described beta-lactam onto polyelectrolyte modified nanosilica [24]. The general isotherm equation [23] is

(4) $$\Gamma =\frac{\Gamma_{\infty }k_{1}C\left(\frac{1}{n}+k_{2}C^{n-1}\right)}{1+k_{1}C\left(1+k_{2}C^{n-1}\right)}\;$$

where $\Gamma_{\infty }$ (mg/g) is the maximum CFX adsorption, $\Gamma_{\infty }$ can be determined from the data of adsorption isotherm at high CFX concentrations, $k_{1}$ (g/mg), and $k_{2}$ (g/mg)^n−1^ are equilibrium constants for first layer adsorption and clusters of n molecules. C (mg/L) denotes the equilibrium concentrations of CFX in solution. 

## 3. Results and Discussion

### 3.1. Characterizations of Protein from Moringa Seeds

The protein extracted from Moringa (MO) seeds was characterized by UV-Vis, FTIR, and HPLC methods.

**Figure 3:**
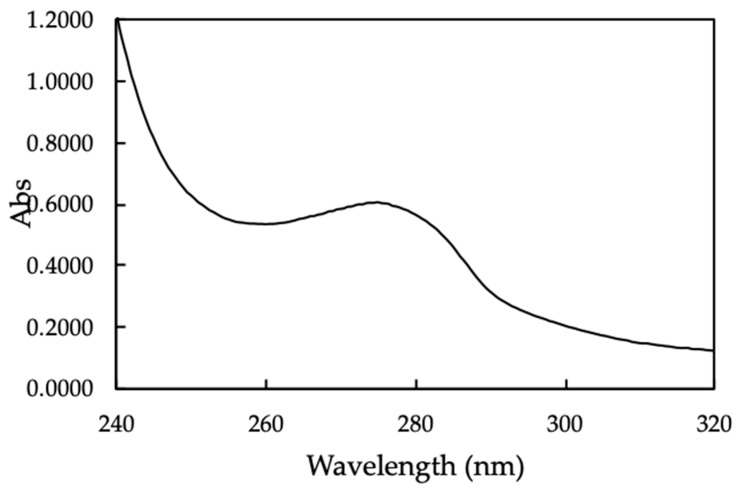
The UV-Vis spectrum of Moringa (MO) seeds protein.

The UV-Vis spectrum of MO seeds protein was indicated in Figure 3.

The UV-Vis spectrum of MO seeds shows a broadened peak with maximum absorbance at 278 nm, which indicate the natural characteristic of protein [33].

**Figure 4:**
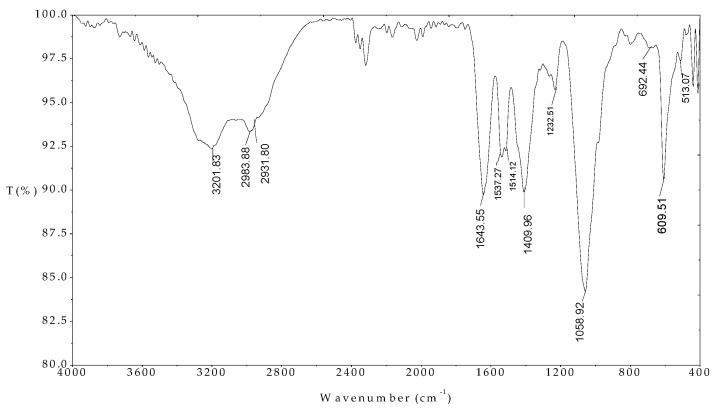
FTIR spectrum of Moringa (MO) seeds protein in the wave number range 400–4000 cm^−1^.

The FTIR spectrum of the MO seeds protein indicated in Figure 4 indicates that the peaks of 1643.35, 1537.27, 1514.12, and 1409.96 cm^−1^ were assigned for C=O stretching amide I, NH amide II, NH amide I bending, and C-H stretching amide III, respectively. These results are in good agreement with previously published paper [34]. In addition, the peaks at 2983.88 and 2931.80 cm^−1^ associated with C-H alkyl in amino acids structures. The FT-IR results confirmed that the presence of amino acids with functional groups are evident. 

The content of existed amino acids in MO seeds protein determined by HPLC-PDA and HPLC-FLD indicated Table 1 shows that the positive amino acids such as Arginine and polar side chain amino acid such as Glutamine have higher content of 4.41% and 5.00%, respectively. These results agree well with protein extracted from MO by Kwaambwa et al. [20]. It implies that high positive charge of amino acids in protein is dominant than negative ones. This results in high isoelectric point (IEP) of MO seeds protein, which is good for surface modification of nanosilica.

### 3.2. Adsorption of Protein onto Nanosilica

#### 3.2.1. Effect of pH on Protein Adsorption 

**Figure 5:**
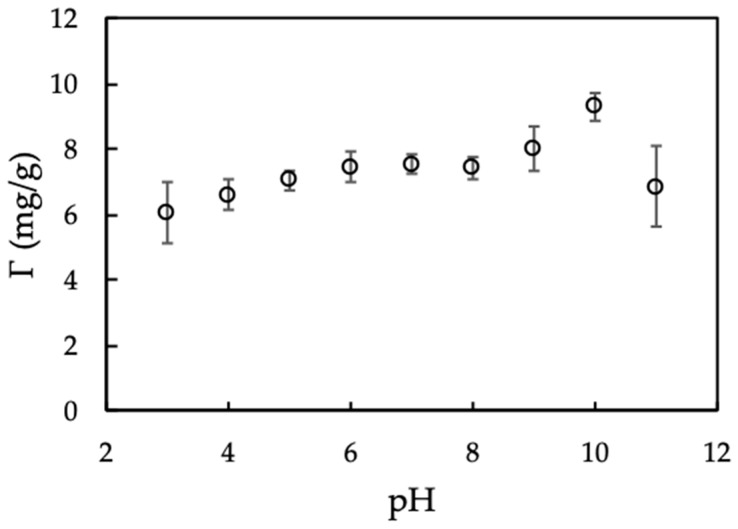
Effect of pH on Moringa seeds protein adsorption onto nanosilica. (*C_i_* (protein) = 100 mg/L, adsorbent dosage 10 mg/mL, 10 mM KCl). Error bars show standard deviations of three replicates.

Figure 5 indicates that MO seed protein adsorption capacity calculated form Equation (2) increases with increasing in the pH range of 3–10 because of an increase of absolute negative charge of nanosilica with increasing pH. From pH 10 to 11, adsorption decreased dramatically due to the dissolution of silica at high pH [35]. Another reason is due to the IEP of protein is about 10 [28], so that protein becomes negative charge at pH > 10. The electrical repulsion force causes the less adsorption between protein and nanosilica. Therefore, pH 10 is optimum to modify nanosilica surface that is kept for further studies.

#### 3.2.2. Effect of Ionic Strength on Protein Adsorption 

Adsorption isotherms at different salt concentrations can demonstrate the effect of ionic strength because the influence of salt strongly induce the electrostatic interactions [36]. 

**Figure 6:**
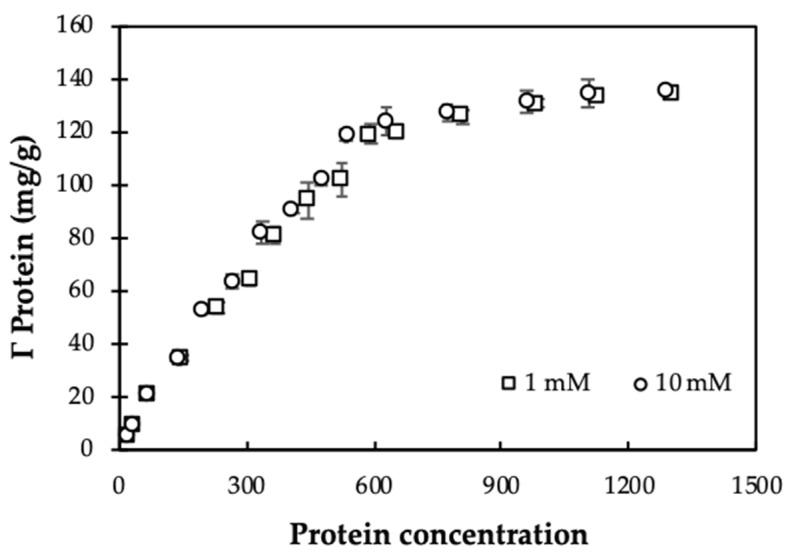
Adsorption isotherms of Moringa (MO) seeds protein onto nanosilica at different KCl concentrations (pH 10). Error bars show standard deviations of three replicates.

Figure 6 shows that at pH 10, adsorption capacities of MO seeds protein onto nanosilica using Equation (2) is independent on ionic strength. Although KCl concentration increased 10 times, the adsorption capacity did not change for different initial concentrations of protein. This trend is similar to adsorption of β-lactoglobulin onto nanosilica at pH 6 [18]. Also, all experimental data are highly repeatable with very small standard deviations of replications. When increasing ionic strength, the number of counter cations increase on the negatively charged nanosilica surface. As a result, a decrease of electrostatic attraction between of protein nanosilica occurred. Nevertheless, protein adsorption onto nanosilica still remains so that other interactions, such as hydrogen bonding, hydrophobic, and lateral interaction can control adsorption [37], demonstrating that protein adsorption on nanosilica is induced by both electrostatic and nonelectrostatic interactions. 

Adsorption of MO seeds protein onto nanosilica at different ionic strengths reaches equilibrium when protein concentration is 2000 mg/L. Thus, the initial protein concentration of 2000 mg/L is suitable for modification of nanosilica surface to enhance removal of CFX.

### 3.3. Adsorptive Removal of Ciprofloxacin (CFX) Using Protein-Modified Nanosilica (ProMNS)

#### 3.3.1. Effect of pH 

**Figure 7:**
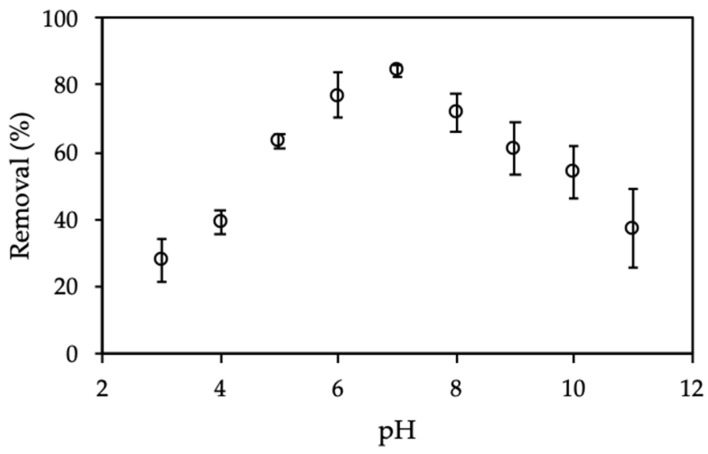
Effect of pH on removal of CFX using protein-modified nanosilica (ProMNS). (C*_i_* (CFX) = 20 mg/L, contact time 90 min, adsorbent dosage 10 mg/mL, 1 mM KCl). Error bars show standard deviations of three replicates.

The solution pH plays the most important role for CFX adsorption onto ProMNS. The removal of CFX calculated by Equation (3) is strongly influenced by pH because of CFX charging behavior and surface charge of ProMNS. Figure 7 shows the effect of initial pH on CFX removal from pH 3 to 11 in 1 mM KCl.

As seen in Figure 7, CFX removal increased dramatically with an increase of pH in the range of 3–7, then the removal decreased significantly from pH 7 to 11. At pH < 6.09 (pK*a*,_1_), CFX has positive charge while at pH higher than 8.62 (pK*a*,_1_), CFX has a negative charge [38]. It implies that at pH 7, CFX is zwitterionic form. However, the maximum removal of CFX is achieved at pH 7, suggesting that CFX adsorption onto ProMNS due to non-electrostatic interactions. On the one hand, at pH < 6.0, CFX removal is low due to the repulsive force between positive CFX species and positively charged ProMNS surface. On the other hand, at pH > 9.0, CFX removal is also low due to the desorption of protein and the dissolution of silica at high pH [39,40]. It should be noted that isoelectric point (IEP) of MO seeds protein is about 9.0 so that the negative form of protein can be taken place at pH > 9.0 [28]. Therefore, solution pH 7.0 is optimum for CFX removal using ProMNS and pH 7.0 was fixed for further investigation. 

#### 3.3.2. Effect of Adsorption Time

**Figure 8:**
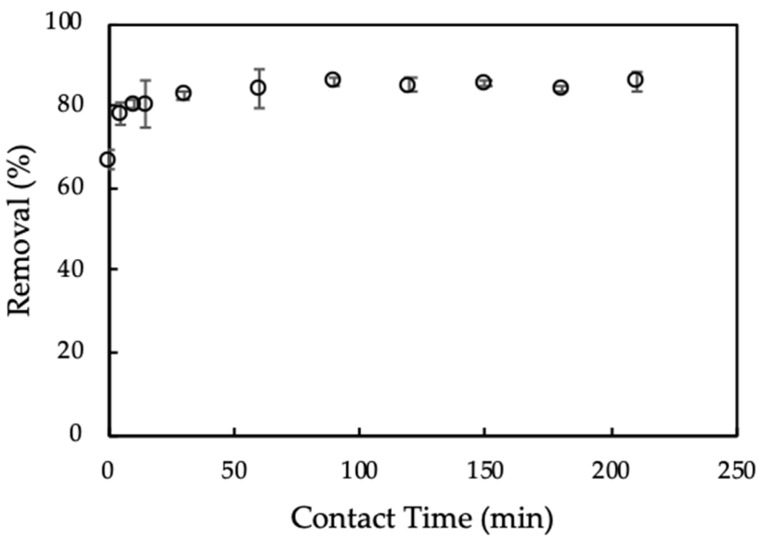
Effect of adsorption time on removal of ciprofloxacin (CFX) using protein-modified nanosilica (ProMNS). C*_i_* (CFX) = 20 mg/L, pH 7.0, adsorbent dosage 10 mg/mL, 1 mM KCl). Error bars show standard deviations of three replicates.

Adsorption time influences the equilibrium process of CFX onto ProMNS. The effect of adsorption time on CFX removal using ProMNS is indicated in Figure 8. As can be seen in Figure 8, CFX removal using Equation (3) increased sharply with increasing adsorption time from 5 min to 30 min. Then, CFX removal still increased when adsorption time increased to 90 min. After 90 min, removal of CFX changes insignificantly, indicating that adsorption equilibrium reaches 90 min. The adsorption time for CFX using ProMNS in this case is faster than CFX adsorption onto tea leaves biochar in which the equilibrium adsorption time is 540 min. Thus, adsorption time 90 min is kept for further study on CFX removal using ProMNS.

#### 3.3.3. Effect of Adsorbent Dosage

**Figure 9:**
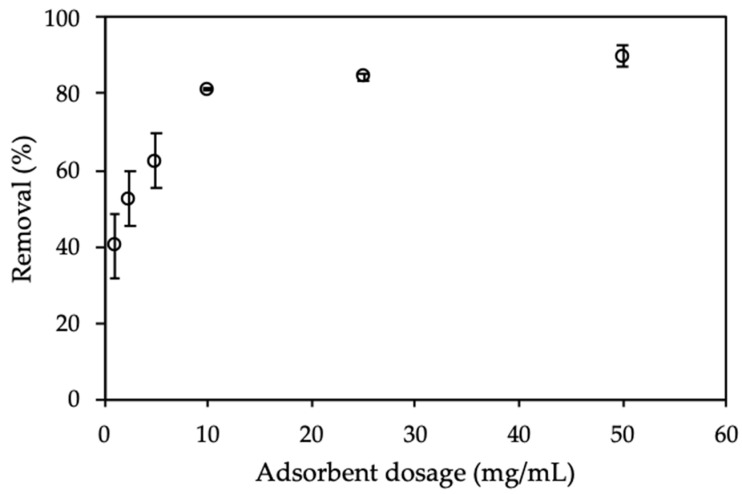
Effect of adsorbent dosage on removal of CFX using protein-modified nanosilica (ProMNS). C*_i_* (CFX) = 20 mg/L, pH 7.0, adsorption time 90 min, 1 mM KCl). Error bars show standard deviations of three replicates.

The adsorbent dosage is effective effect on the adsorption process, because it influences the total surface area and charge density of the adsorbent [4,41,42,43]. The dosage of ProMNS varied from 1.0 to 50.0 mg/mL (Figure 9).

Figure 9 shows that CFX removal using ProMNS calculated by Equation (3) increased dramatically with an increase of adsorbent dosage from 1.5 to 10 mg/mL. This phenomenon is due to a large number of binding sites for adsorption or the enhancement of specific surface area with increasing adsorbent amount [44]. Nevertheless, when increasing adsorbent dosage higher than 10 mg/mL, CFX removal changed insignificantly. The error bar showing the deviations of three replicates with 10 mg/mL is also smallest comparing with other dosages. It implies that an adsorbent dosage of 10 mg/mL is suitable for CFX removal through adsorption technique using ProMNS. 

#### 3.3.4. Effect of Ionic Strength

**Figure 10:**
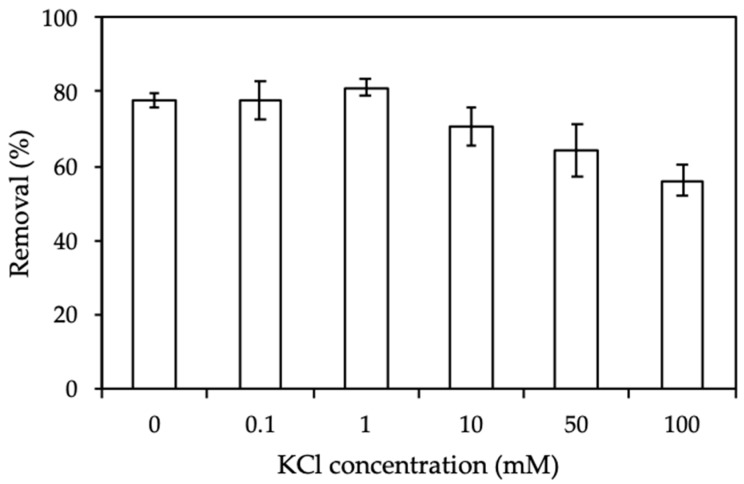
Effect of ionic strength on removal of CFX using protein-modified nanosilica (ProMNS). (*C*_i_ (CFX) = 20 mg/L, pH 7.0, adsorption time 90 min, adsorbent dosage 10 mg/mL). Error bars show standard deviations of three replicates.

Ionic strength influences the electrostatic interaction between the positively charged ProMNS surface and CFX molecular. Figure 10 shows the results of CFX removal using ProMNS calculated by Equation (3) at KCl concentration from 0 to 100 mM. As can be seen, at *C*_KCl_ < 1 mM, CFX removal change slightly. However, CFX removal decreases dramatically when increasing salt from 1 to 100 mM KCl. It implies that the electrostatic interaction between CFX molecular and ProMNS is screened with an increase of KCl concentration. As a result, CFX removal using ProMNS is highly influenced by the screening of electrostatic force. The effect of ionic strength is investigated and discussed in detail by adsorption isotherms described below.

### 3.4. Adsorption Isotherms of CFX onto Protein-Modified Nanosilica (ProMNS)

**Figure 11:**
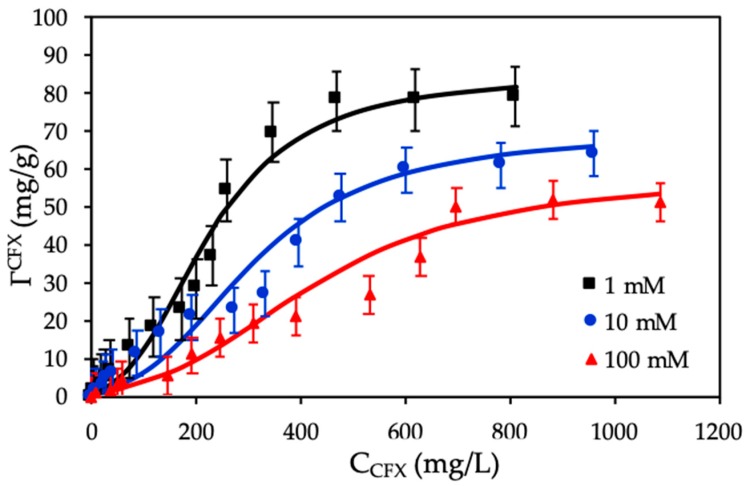
Adsorption isotherms of CFX onto protein-modified nanosilica (ProMNS) at different KCl concentrations. Points are experimental results and solid lines are fitted by two-step adsorption model. Error bars show standard deviations of three replicates.

Adsorption isotherms of CFX onto ProMNS were achieved with initial concentrations of antibiotic in the range of 20–1600 mg/L. The two-step adsorption model was used to fit adsorption isotherms of CFX onto ProMNS (Figure 11).

Figure 11 shows that the higher KCl concentration is, the lower adsorption capacity is. At different initial CFX concentrations, adsorption at 100 mM KCl is always lower than that at 10 mM and 10 mM is lower than 1 mM. The CFX adsorption increased with decreasing KCl concentration due to a decrease of various cations K^+^ on the ProMNS surface with positive charge. As a result, an increase of the electrostatic attraction was obtained. Figure 11 also indicates that adsorption isotherms of CFX onto ProMNS at three KCl concentrations could be reasonably represented by two-step adsorption model with Equation (4) using the fitting parameters in Table 2. The fitting values for *k*_1_ and *Γ*_CFX_ were calculated from the Langmuir isotherms while other fitting parameters (*k*_2_ and *n*) at different salt concentration are calculated by trials and error method using OriginPro 8. Although some experimental points have quite high deviations compared with modeling, almost experimental values matched the calculated one from the model. In this case, the deviations are higher than beta lactam cefixime adsorption onto strong polycation, polydiallyldimethylammonium chloride (PDADMAC) modified nanosilica rice husk because the charging properties of protein is less than that of PDADMAC. However, the two-step model used in the present study to represent adsorption isotherms of CFX onto ProMNS is much better than the fit of cellulose-based polymer adsorption isotherms onto cotton fibers [45]. It can be seen that all error bars show standard deviations of three replicates are suitable and close to the solid lines fitted by two-step adsorption model. The Table 2 shows that the value of *k*_1_ decreased with increasing KCl concentrations from 1 to 100 mM while the *k*_2_ values are constant for 1 and 10 mM and slightly decrease for 100 mM. Furthermore, the values of *n* increased slightly with increasing ionic strength for CFX adsorption. It implies that *k*_1_ and *n* are useful parameters to evaluate the influence of ionic strength on CFX adsorption onto ProMNS. 

Table 2 also shows that the maximum adsorption capacity of CFX reaches to 85 mg/g at 1 mM KCl. Adsorption capacity in our case is highest compared with different adsorbents for CFX removal [46], demonstrating that ProMNS is a novel, eco-friendly material for CFX removal from aqueous solution due to the low cost of nanosilica synthesized from rice husk and natural protein extracted from MO seeds.

### 3.5. Adsorption Mechanisms of CFX onto Protein-Modified Nanosilica (ProMNS)

In this section, adsorption mechanism of CFX onto ProMNS are discussed in detail on the basis of the surface charge change by ζ potential, the change in surface functional group by FT-IR, and adsorption isotherm of CFX onto ProMNS. 

**Figure 12:**
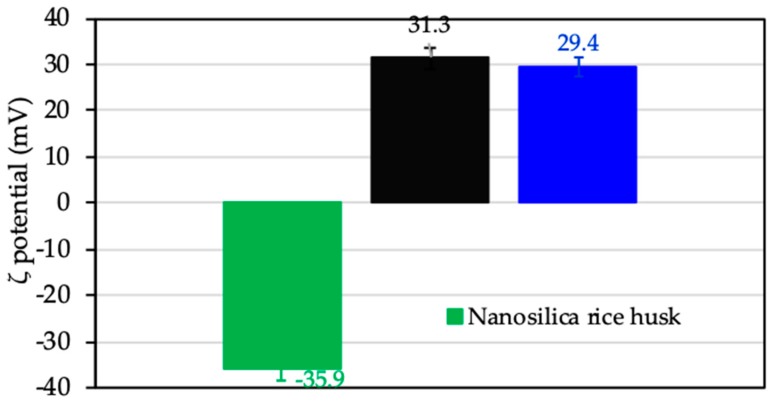
The ζ potential of nanosilica rice husk, protein-modified nanosilica (ProMNS), and ProMNS after CFX adsorption. Error bars show the standard deviations of three replicates.

Surface charge change by monitoring ζ potential calculated by Equation (1) before and after adsorption of protein was thoroughly studied [18,47]. In the present study, the ζ potential was used to evaluate charging behavior of nanosilica rice husk without adsorption, after protein adsorption, and CFX adsorption to suggest the adsorption mechanism of CFX onto ProMNS. Figure 12 shows that charge reversal occurred after modification of nanosilica by protein adsorption. Nanosilica with negative ζ potential −35.9 mV changed to positive (ζ = 31.3 mV) after modification with protein (ProMNS). However, after CFX adsorption at pH 7.0 (pKa_1_ < pH < pK*a_2_*), with the zwitterionic species of CFX, a decrease in positive charge of ProMNS is observed. These results are in good agreement with the effect of pH on the CFX removal (Section 3.3.1).

**Figure 13:**
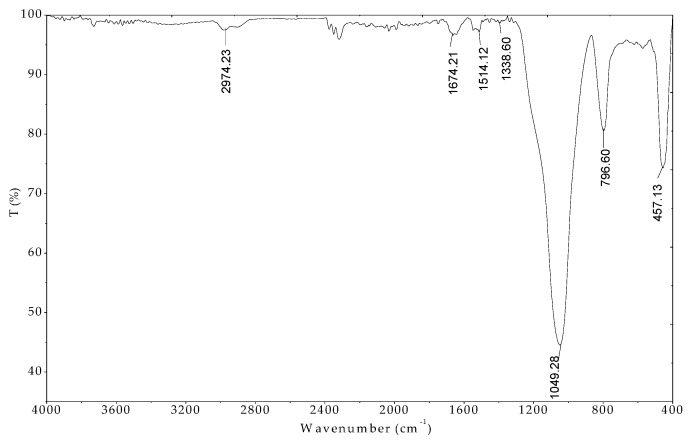
FTIR spectrum of nanosilica with protein modification after Ciprofloxacin (CFX) adsorption in the wave number range 400–4000 cm^−1^.

The FT-IR is useful tool to evaluate the surface functional groups in adsorption technique [48]. Figure 13 shows the spectrum of protein-modified nanosilica (ProMNS) after CFX adsorption in the range of wavenumber 400–4000 cm^−1^.

The FT-IR spectrum of nanosilica synthesized from rice husk [24]. The surface modification of nanosilica by protein adsorption (ProMNS) was achieved in the presence of the peaks with the wavenumbers of 1645.28, 1539.20, and 1514.12 cm^−1^ (not shown here). Nevertheless, after CFX adsorption, only the peak of 1514.12 cm^−1^ assigned for NH amide I bending occurred. In addition, a small peak appeared at 1338.60 cm^−1^ indicating aromatic nitro compound of CFX in the FT-IR spectra of ProMNS after CFX adsorption [49]. Also, the characteristics of stretching vibration C=O at 1045.42 cm^−1^ and phenolic C-OH stretch at 1267.23 cm^−1^ of molecular CFX disappear after adsorption indicate that CFX adsorption occurs onto ProMNS surface by carboxyl groups. Therefore, the less positive charge of ProMNS was obtained. These results agree with the changes in surface charge of ProMNS after CFX adsorption are further in accordance to adsorption isotherms presented above. The driving force inducing the CFX adsorption is mainly by electrostatic attraction between negative CFX species with positively charged ProMNS surface. 

### 3.6. Adsorption Kinetics of CFX onto Protein-Modified Nanosilica (ProMNS)

The adsorption kinetics of CFX onto ProMNS were carried out at the three initial CFX concentrations of 20, 200, and 1200 mg/L from 0 to 210 min. 

The pseudo-second-order was used to predict the adsorption kinetic.

(5) $$\frac{t}{q_{t}}=\frac{1}{k_{k}\cdot q_{e}^{2}}+\;\frac{1}{q_{e}}t\;$$

where *q_e_* and *q_t_* (mg/g) are adsorption capacity of RhB onto SML at equilibrium and time *t*, respectively; *k_k_* (g/mg·min) is reaction rate constant of pseudo-second-order adsorption kinetic.

**Figure 14:**
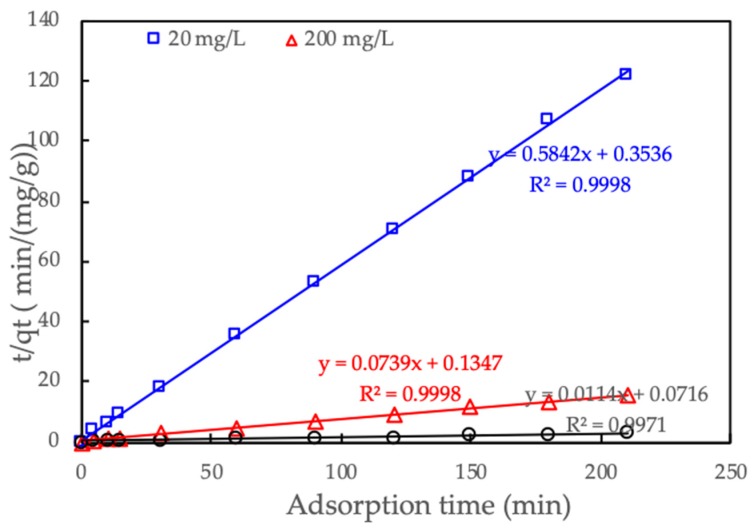
Adsorption kinetics of CFX onto protein-modified nanosilica (ProMNS) for three initial CFX concentrations. Points are experimental results and solid lines are fitted by pseudo-second-order model.

Figure 14 shows that the pseudo-second-order fitted experimental kinetic data for three concentrations of CFX concentrations very well. All excellent of R^2^ (greater than 0.997) indicates that the adsorption kinetics of CFX onto ProMNS are in good agreement with the pseudo-second-order model. These results are similar to CFX adsorption onto biocomposite fibers of graphene oxide/calcium alginate [50] in which pseudo-second-order achieved the best fit comparing with other kinetic models. 

### 3.7. Adsorptive Removal of CFX Using Nanosilica without and with Surface Modification by Protein 

**Figure 15:**
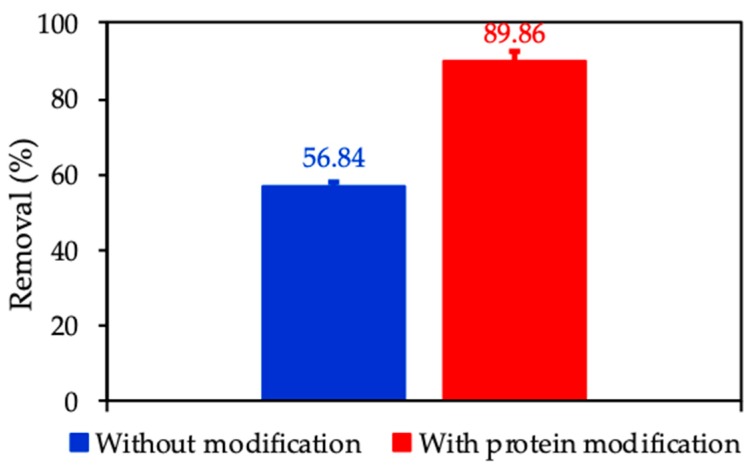
Removal of CFX using nanosilica without and with protein modification. Error bars show standard deviations of three replicates.

To emphasize the role of surface modification with protein adsorption, we compared the removal of CFX using nanosilica without and with protein. Figure 15 indicates that CFX removal with an initial concentration of 20 mg/L in 1 mM KCl using the same adsorbent dosage of 10 mg/mL increases about 1.6 times from 56.84% to 89.85% with protein modification. The enhancement of CFX removal due to the increase of electrostatic attraction between the molecular CFX and the positively charged ProMNS surface. This implies that ProMNS is better than nanosilica synthesized from rice husk without any modification in term of CFX removal.

### 3.8. Comparison of the Effectiveness of ProMNS and Other Adsorbents for Removal of CFX

Recently, many studies have reported various adsorbents for CFX removal. However, adsorptive removal of CFX using ProMNS has not been investigated. In addition, the ProMNS in this study has highest removal efficiency and capacity compared to other adsorbents (Table 3). Another feature is that ProMNS is a green and low-cost adsorbent because MO seeds protein is natural product while rice husk is agricultural sub-product. This implies that ProMNS is not only a new material, but also an eco-friendly material for antibiotics removal from aqueous solutions.

**Figure 16:**
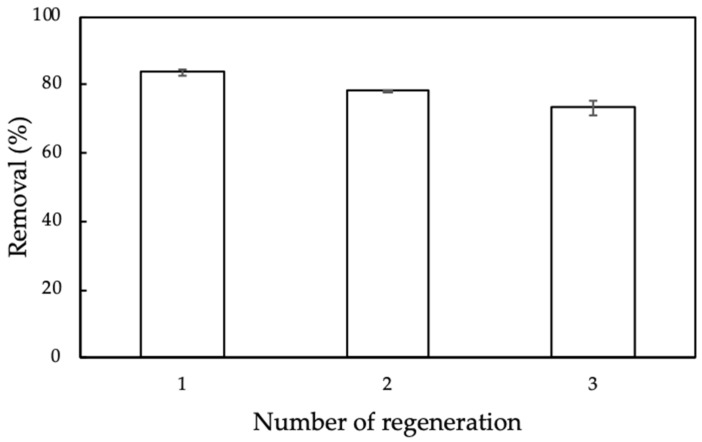
Removal of CFX using protein-modified nanosilica (ProMNS) after three regenerations. Error bars show standard deviations of two replicates.

Although nanosilica synthesized from rice husk with surface modification with protein extracted from MO seeds is low-cost adsorbent, the adsorption cycles needed to evaluate the reuse potential and stability of ProMNS. The regeneration of ProMNS by using 0.2M HCl was repeated three times. Figure 16 shows the CFX removal using ProMNS after three cycles. Although the removal was decreased, it was still higher than 73% after three regenerations. The error bars show the standard deviations for all cycles are very small, demonstrating that ProMNS is novel and reusable adsorbent. 

### 3.9. Removal of CFX from Hospital Wastewater Using Protein-Modified Nanosilica (ProMNS)

The removal of antibiotics from actual wastewater sample is important to understand the implication for real system. We used ProMNS to removal CFX from hospital. An actual wastewater sample, which was selected from a big hospital in Hanoi, Vietnam was used to attempt to remove CFX using ProMNS under optimum conditions. An actual hospital wastewater sample remained in a dark bottle in a refrigerator before carrying out removal experiment in our laboratory. 

**Figure 17:**
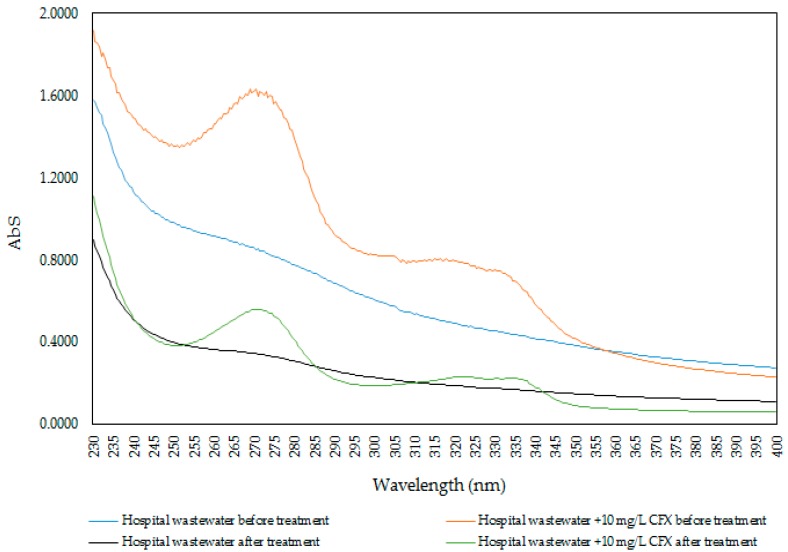
UV-Vis spectrum of CFX of the actual hospital wastewater samples before and after treatment using protein-modified nanosilica (ProMNS).

For CFX removal with an actual sample, the experiments were conducted within 48 h. The optimum conditions for CFX removal using ProMNS were pH 7, adsorption time 90 min, and adsorbent dosage 10 mg/mL. Due to the very low CFX residual concentration to evaluate the performance, a 10 mg/L CFX was added into an actual sample to evaluate removal efficiency. Figure 17 indicates the UV-Vis spectra of CFX in the actual hospital wastewater samples before and after treatment using ProMNS. For the actual wastewater sample without addition, the spectrum is dramatically decreased, indicating that not only CFX, but also other organic pollutants were treated. In addition, the specific wavelength of 272 nm for actual sample with 10 mg/L CFX decreases sharply. By basic calculation at the maximum absorbance, the removal efficiency of CFX using ProMNS was achieved about 70%. This results again demonstrate that ProMNS is a novel, eco-friendly adsorbent for antibiotics removal from hospital wastewater. 

## 4. Conclusions

The removal of emerging pollutant, antibiotic ciprofloxacin (CFX) using the Moringa (MO) seeds protein-modified nanosilica rice husk (ProMNS) was systematically investigated in the present study. Protein extracted from MO seeds had 18 amino acids with high purity, which was characterized by FTIR, UV-Vis, and HPLC. Adsorption of protein onto nanosilica was induced by both electrostatic and non-electrostatic interaction at pH 10. The CFX removal increased about 1.6 times using nanosilica after surface modification with protein. Optimum conditions for CFX removal from aqueous solution using ProMNS were thoroughly studied and found to be pH 7.0, adsorption time 90 min, adsorbent dosage 10 mg/mL, and ionic strength 1 mM KCl. Under optimum conditions, the maximum adsorption capacity reached 85 mg/g, which is much higher than many common adsorbents. Adsorption isotherms of CFX onto ProMNS at three KCl concentrations were fitted well by a two-step adsorption model while adsorption kinetics are in good agreement with the pseudo-second-order model. Adsorption of CFX onto ProMNS was mainly controlled by electrostatic attraction between anionic species of CFX and positively charged ProMNS surface. After three regenerations, the CFX removal was still higher than 73% and a high CFX removal of about 70% was achieved with an actual hospital wastewater when using ProMNS. We indicate that ProMNS is an excellent and new eco-friendly material to remove antibiotics from hospital wastewater.

## Figures and Tables

**Table 1:** The amino acids contents in protein extracted from Moringa (MO) seeds.

Amino Acid	Content (%)	Standard Deviation
Histidine	0.70	0.02
Serine	0.67	0.08
Glycine	1.14	0.19
Arginine	4.41	0.38
Aspartic	0.68	0.21
Glutamine	5.00	0.62
Threonine	0.56	0.07
Alanine	0.88	0.12
Proline	1.55	0.12
Cystine	0.38	0.04
Lysine	0.35	0.10
Tyrosine	0.57	0.01
Methionine	0.52	0.01
Valine	0.92	0.07
Isoleucine	0.91	0.11
Leucine	1.49	0.13
Phenylalanine	1.40	0.03
Tryptophan	0.36	0.03
Total	22.10	0.20

**Table 2:** The fitting parameters for CFX adsorption onto protein-modified nanosilia (ProMNS) at different KCl concentrations.

*C*_KCl_ (mM)	Γ_CFX_ (mg/g)	*k*_1_ (g/mg)	*k*_2_ (g/mg) ^n−1^	*n*
1	85	10.0 × 10^2^	4.0 × 10^6^	3.0
10	70	9.0 × 10^2^	4.0 × 10^6^	3.1
100	58	7.0 × 10^2^	3.5 × 10^6^	3.2

**Table 3:** Adsorption capacity and removal efficiency of protein-modified nanosilica (ProMNS) and other absorbents for ciprofloxacin (CFX) removal.

Adsorbent	Adsorption Capacity (mg/g)	Removal Efficiency (%)	References
Graphene oxide/calcium alginate biocomposite	39.06	NI	[50]
Activated sludge	10.87	59	[51]
Kaolinte	7.95	50	[52]
Nanoscale zerovalent iron (nZVI) -Cu	NI	81.6	[53]
Silica nanoparticle	30	78	[54]
Hydrous oxides of Al (HAO)	13.6	NI	[55]
ProMNS	85	89.86	This study

NI: no information.
